# Paratesticular Leiomyosarcoma: The First Reported Case From Sri Lanka

**DOI:** 10.7759/cureus.107536

**Published:** 2026-04-22

**Authors:** Nona Shalisha Sallay, Tharushi Dayaratna, H L D S Ariyaratne

**Affiliations:** 1 General Surgery, Colombo South Teaching Hospital, Nugegoda, LKA

**Keywords:** high inguinal orchidectomy, leiomyosarcoma, para testicular tumor, scrotal mass, soft tissue sarcoma

## Abstract

Paratesticular leiomyosarcoma is an uncommon urological malignancy, with only a limited number of cases reported globally. This report describes the first documented instance in Sri Lanka. The patient was a 68-year-old male who presented with a painful mass on the left side of the scrotum. A high inguinal orchidectomy was performed, and histopathological analysis confirmed a diagnosis of paratesticular leiomyosarcoma. This case underscores the necessity of considering rare malignancies in the differential diagnosis of scrotal masses. It further highlights the critical role of imaging and histopathological evaluation in achieving an accurate diagnosis.

## Introduction

Accounting for only 1-2% of all urologic malignancies, soft-tissue sarcomas represent a rare subset of tumors within the genitourinary tract [1]. Para-testicular leiomyosarcomas are mesenchymal malignancies with smooth muscle differentiation arising from the testicular tunica, epididymis, or spermatic cord. We report the first documented case of para-testicular leiomyosarcoma diagnosed and treated in Sri Lanka.

## Case presentation

A 68-year-old male presented with a progressively enlarging, tender swelling of the left hemi-scrotum over a period of 9 months. There were no associated lower urinary tract symptoms or constitutional symptoms. His past medical and surgical history was uneventful. Physical examination revealed a solid, tender, 5x4 cm swelling, palpable separately from the left testis. An ultrasound scan of the scrotum identified a solid 5.5x4.8x4.3 cm³ hypoechoic septated mass in the left hemiscrotum. The origin of the mass was unclear, and the epididymis and testis were identified separately from the mass. Lactate dehydrogenase (LDH; 159 U/L) and β-human chorionic gonadotropin (β-hCG; <1.2 IU/L) levels were within normal limits.

**Figure 1:**
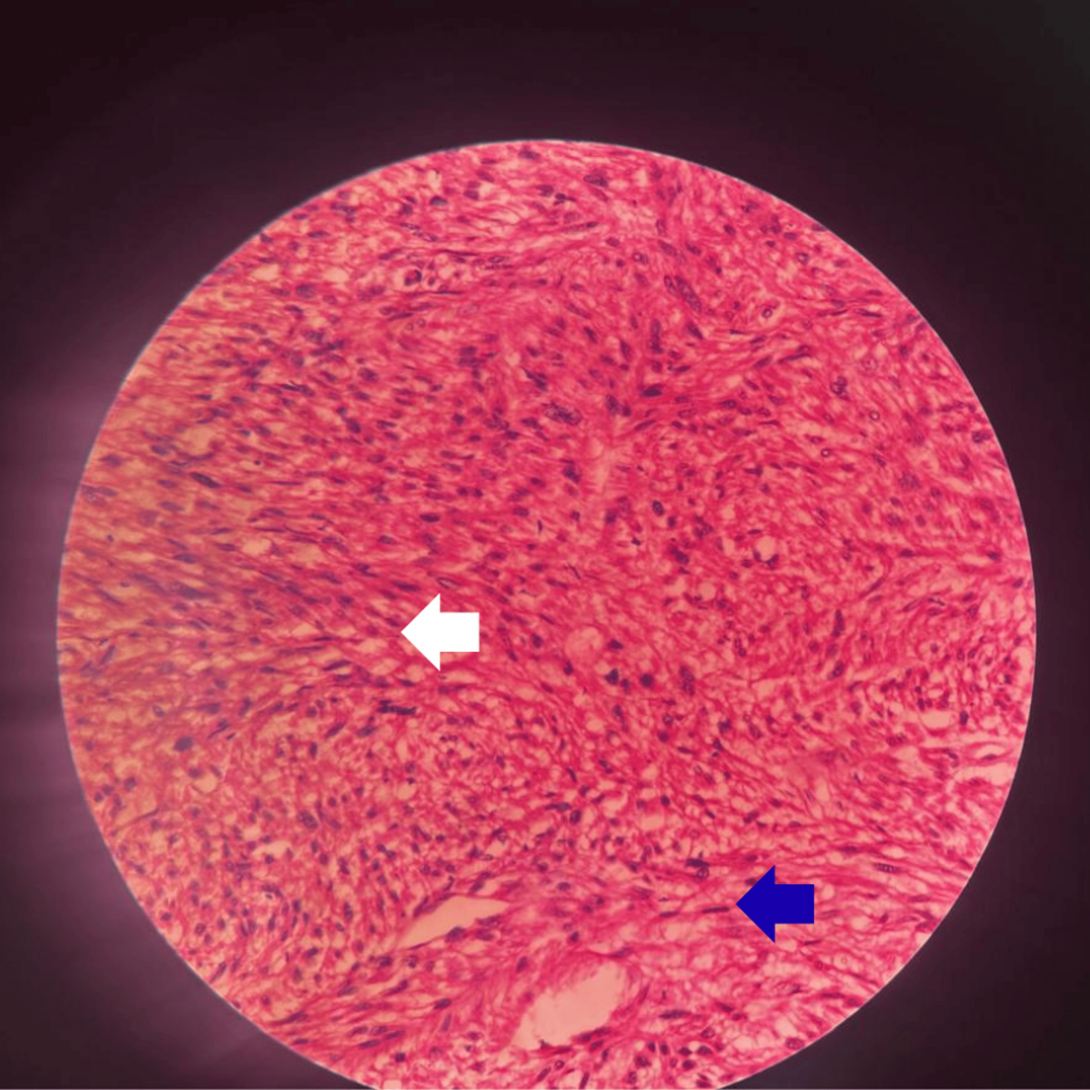
Histopathological findings of the malignant spindle cell tumor (Hematoxylin and Eosin stain, 40x magnification) The section reveals a densely packed spindle cell population arranged in intersecting bundles and fascicles (white arrow). Marked nuclear pleomorphism and characteristic "cigar-shaped" nuclei (blue arrow) are evident throughout the specimen, consistent with a high-grade malignant process.

The patient underwent an elective left-sided high inguinal orchidectomy. The specimen consisted of the left testis with coverings, epididymis, spermatic cord, and a mass loosely attached to the spermatic cord. The solid mass measured 6x5x4 cm³, and the cut surface was tan. Histology reported a malignant tumor composed of fascicles of spindle-shaped cells (Figure 1). These cells showed prominent nuclear atypia. Mitotic activity amounted to 2-3/10 HPF. Prominent infarct-type necrosis was present. Additionally, necrotic foci with admixed karyorrhectic debris were noted. The tumour was located within the paratesticular soft tissue, outside the testis and epididymis. The testis, epididymis, and spermatic cord appeared histologically normal, with no direct tumour infiltration.

The surgical margin of the spermatic cord showed no evidence of tumor involvement. Immunohistochemical staining demonstrated strong and diffuse positivity for smooth muscle actin and desmin. Lymphovascular invasion was absent. The tumour was graded as FNCLCC (Fédération Nationale des Centres de Lutte Contre le Cancer) Grade 2 leiomyosarcoma. Contrast-enhanced CT of the chest, abdomen, and pelvis showed no metastatic disease. The patient is currently under regular follow-up with clinical surveillance scheduled at six-month intervals.

## Discussion

Epidemiology and classification

Soft-tissue sarcomas that originate in the genitourinary system are exceedingly uncommon, representing just 1-2% of all cancers in the urinary and genital tracts [1-4]. Among adult neoplasms, soft-tissue sarcomas account for only 1% of cases [3]. Primary paratesticular tumors themselves are infrequent, comprising 7-19% of all intrascrotal tumors [5,6]. Paratesticular tumors typically arise from three distinct regions: the epididymis, the testicular tunics, or the spermatic cord [1,5]. Leiomyosarcoma is frequently identified in this area, accounting for nearly one-third (19-32%) of all paratesticular malignancies, surpassed only by liposarcoma in frequency [4,6,7].

Pathogenesis and clinical presentation

The anatomical derivation of paratesticular leiomyosarcoma is diverse, potentially arising from the scrotal skin, dartos muscle, epididymis, spermatic cord, or seminiferous tubules. Pathologically, these are categorized into cutaneous forms (originating from the dartos or arrector pili muscles) and subcutaneous variants, which stem from the vascular or genital smooth muscle [3]. While paratesticular sarcomas generally demonstrate a bimodal age peak (16-20 and >60 years), LMS is specifically characterized by a higher incidence during the sixth and seventh decades of life [4,6]. The typical clinical feature is a unilateral, often asymptomatic, intrascrotal swelling, though an associated hydrocele may be present [4,6,8]. Less common manifestations include acute scrotal pain resulting from intra-tumoral hemorrhage or infarction-type necrosis [4].

Our patient was a 68-year-old male who presented with a progressively enlarging, unilateral, tender hemiscrotal mass, which is consistent with the recognized age distribution and an uncommon symptomatic presentation likely related to the necrotic changes identified on histology.

Diagnostic challenges and imaging

The preoperative diagnosis of paratesticular leiomyosarcoma remains challenging due to its rarity and nonspecific clinical and radiological features. These tumors typically present as painless, slow-growing scrotal masses that closely mimic benign conditions such as hydrocele, epididymitis, or other paratesticular lesions [9,10]. Physical examination findings are non-specific, and serum tumour markers, including beta-human chorionic gonadotropin, alpha-fetoprotein, and LDH, are usually within normal limits, further reducing clinical suspicion of malignancy.

Similarly, in our case, serum tumor markers were within normal limits, and preoperative imaging demonstrated a solid extratesticular mass but could not definitively establish the histological diagnosis.

Ultrasound remains the initial imaging modality for evaluating scrotal masses and for distinguishing intratesticular from paratesticular lesions. However, most sarcomas cannot be reliably differentiated from one another on sonographic features alone, except liposarcomas [6]. For further characterization, particularly to assess local extension and detect possible regional or distant disease, cross-sectional imaging, such as MRI and CT, is used [4]. Despite these investigations, radiological findings are often non-specific, and definitive diagnosis is ultimately established only after histopathological examination of the excised specimen, which remains the gold standard [9,10].

The diagnostic difficulty is further compounded by the tumor’s rarity and its overlap with other benign and malignant mesenchymal lesions. Varzaneh et al. highlight that its uncommon nature and ability to mimic benign conditions, such as chronic epididymitis, contribute significantly to diagnostic uncertainty, often necessitating immunohistochemical analysis for accurate tumour classification and confirmation of origin [11]. In addition, sonographic overlap between benign leiomyomas and malignant leiomyosarcomas may result in equivocal preoperative interpretation, while misleading clinical histories, such as trauma or presumed infection, can further delay diagnosis [12]. These factors underscore the importance of maintaining a high index of clinical suspicion and considering early radical surgical intervention when a solid extratesticular mass is identified [11,12].

Histopathology and management

The definitive diagnosis is achieved through histopathological analysis. The hallmark microscopic features observed in this case include spindle cells with eosinophilic cytoplasm and the classic blunt-ended or 'cigar-shaped' nuclear morphology indicative of leiomyosarcoma. While low-grade leiomyosarcoma carries a favorable prognosis, high-grade variants are aggressive, frequently metastasizing, and associated with increasing mortality rates [6,13].

In our patient, additional adverse histological features included nuclear atypia, mitotic activity of 2-3/10 HPF, and tumor necrosis. The tumor was reported as FNCLCC Grade 2, indicating intermediate-grade biological behaviour with recognized metastatic potential and a need for structured long-term surveillance.

Given the rarity of the disease, with only approximately 110 cases reported globally, treatment strategies are largely based on retrospective evidence [8,14]. While the standard of care is a high inguinal orchidectomy, certain clinical scenarios necessitate a wider radical resection to address potential involvement of the surrounding tissue [3,14].

Consistent with current recommendations, our patient underwent high inguinal orchidectomy with negative spermatic cord margins and no radiological evidence of metastatic disease on postoperative staging CT.

## Conclusions

The scarcity of paratesticular leiomyosarcoma does not diminish its clinical significance as an aggressive urological malignancy. This case serves as a reminder that solid, slow-growing scrotal masses in older men should be evaluated with suspicion for mesenchymal tumors, as early identification is paramount to effective management. Because clinical examination and imaging often fail to differentiate these lesions from more common benign conditions or germ cell tumors, a systematic diagnostic approach is essential. A definitive diagnosis relies heavily on meticulous histopathological examination and a comprehensive immunohistochemistry panel to confirm smooth muscle origin and exclude other spindle cell mimics.

To optimize margin status and prevent local recurrence, high inguinal orchidectomy is the primary surgical choice. Despite the uncertainty surrounding auxiliary treatments like radiotherapy, the potential for hematogenous or lymphatic dissemination means that vigilant, long-term monitoring is a non-negotiable part of the care plan. Documentation of this first reported case from Sri Lanka contributes to the global oncological database, highlighting the need for clinician awareness in diverse geographical regions to ensure early detection and optimized surgical intervention.
